# ASSOCIATIONS BETWEEN NURSING HOME WORKPLACE SAFETY CULTURE AND PATIENT SAFETY CULTURE

**DOI:** 10.1093/geroni/igae098.3237

**Published:** 2024-12-31

**Authors:** Brandon Hesgrove, Emily Tyler, Naomi Yount, Joann Sorra, Susan Hassell

**Affiliations:** Westat, Rockville, Maryland, United States; Westat, Rockville, Maryland, United States; Westat, Rockville, Maryland, United States; Westat, Rockville, Maryland, United States; Westat, Rockville, Maryland, United States

## Abstract

An influential report from the Institute for Healthcare Improvement purports that a strong workplace safety culture is a precondition of a strong culture of safety in healthcare. However, existing empirical evidence is limited. We used data from a 2023 pilot test of the Workplace Safety Supplemental Item Set administered with the Surveys on Patient Safety Culture (SOPS) Nursing Home Survey to examine associations between all 9 workplace safety culture and all 14 resident safety culture measures. Data were obtained from 2,515 staff (40% response rate) in 48 nursing homes across 20 states. Associations were estimated using multiple linear regressions, where, because of strong multicollinearity, the dependent variable for each regression was one patient safety culture measure and the independent variables were one workplace safety culture measure, nursing home bed size, and ownership. The analysis was at the nursing home level and nursing home percent positive scores were calculated as the percentage of staff that answered with the two most positive response options (out of five). 123 out of 126 associations were statistically significant (p < 0.05). The two workplace safety culture measures most highly associated with resident safety culture were Management Support for Workplace Safety (mean β = 0.78) and the Overall Rating on Workplace Safety for Staff (mean β = 0.73). The very strong associations between resident and workplace safety culture measures imply that the two are interconnected and improving a culture of safety for nursing home staff will also improve safety for residents.

